# Aging Characterizations of Modified Asphalt Binders Based on Low Field Nuclear Magnetic Resonance (LF-NMR)

**DOI:** 10.3390/ma15228224

**Published:** 2022-11-19

**Authors:** Lili Wang, Xinsheng Li, Junan Shen, Jing Li, Wei Wang

**Affiliations:** 1 Suzhou Sanchuang Pavement Engineering Co., Ltd., Suzhou 215011, China; 2 Jiangsu Provincial Center of Ecological Road Technology Industrialization and Research, Suzhou University of Science and Technology, Suzhou 215011, China; 3 Department of Civil Engineering and Construction, Georgia Southern University, Statesboro, GA 30458, USA; 4 School of Civil Engineering, Chongqing Jiaotong University, Chongqing 400074, China

**Keywords:** low field nuclear magnetic resonance (LF-NMR), SBS, CRM, modified asphalt, aging

## Abstract

Styrene-butadiene-styrene block copolymer (SBS) and crumb rubber modifier (CRM) are commonly used modifiers to modify asphalt binders. The aging of modified asphalt binders is an important factor affecting their performance. In this paper, the effects of the two modifiers (i.e., SBS, CRM) on the aging of modified asphalt binders were studied by using low field nuclear magnetic resonance (LF-NMR) technology and dynamic shear rheological (DSR) tests. Test results showed that when T2, a parameter of relaxation time from NMR test, was within 2.2 milliseconds, the relaxation peak of both modified and unmodified asphalt binders tested showed two peaks (i.e., “M” shape), and when it was greater than 2 milliseconds, extra peaks appeared only in the modified asphalts. These extra peaks gradually disappeared with increased aging; the modifiers carried signal intensities of their own. The addition of a modifier changes the law of “the greater the viscosity of asphalt, the shorter the T2 relaxation time”. With the aging process, the normalized peak area (NPA) from NMR decreased, and rutting resistance factor from DSR increased. However, the NPA of modified asphalt increased after the PAV test, which may be related to the change of H semaphore. The rheological properties of the asphalt binders before and after aging were well-correlated with the NPA of T2.

## 1. Introduction

As the demand to have high quality asphalt mixtures for pavements has increased, the research on asphalt modification has also increased. SBS is one of the most widely used asphalt modifiers; studies have shown that when base asphalt is mixed with SBS that will absorb some components of the base asphalt and expand, an SBS network will be formed in the base asphalt [1]. The modification will improve the properties and aging resistance of modified asphalt binders, and consequently the performance of the asphalt mixtures with a modified binder [2,3,4,5,6]. CRM, another common modifier, can effectively improve the anti-rutting and anti-cracking performance of modified asphalts. According to life cycle economic analysis, the CRM asphalt mixture is one of the most cost-effective modified asphalt mixtures, and it is better than traditional mixtures [7]. It is important to accurately characterize the effect of the modifiers on modified asphalt binders. Therefore, many modern test methods have been invented, such as atom force microscope (AFM), Fourier transform infrared spectroscopy (FTIR), and fluorescence microscopy. However, these methods will cause different degrees of damage to asphalt before measurement, so a fast and non-destructive test method is needed to study the properties of modified asphalt.

LF-NMR is widely used in life science, agricultural food, energy exploration, and other industries due to its advantages of it being fast and accurate, and there is no damage to the internal structure of samples. It is increasingly used in the study of aged and rejuvenated asphalt binders, such as in the analysis of viscosity. The concept of this analysis is based on the good relationships between asphalt viscosity and lateral relaxation time T2 and relative hydrogen index (RHI) measured by NMR [8].

LF-NMR can measure two parameters of asphalt binders: amplitude and relaxation time of the hydrogen nucleus [9]. The relaxation time includes the transverse and longitudinal components of T2 and T1. T2 was adopted for analysis in this study. T2 is also interpreted as the attenuation of the transverse component of magnetization relative to the asphalt hydrogen nucleus. For the high viscosity of asphalt binders, the relaxation time is related to the volume movement of molecules [10]. In general, an increase in sample viscosity results in a decreased T2 time because the spin echo decay (SED) of T2 is faster in a high-viscosity component than in a low-viscosity component. In addition, with the extension of aging time and the increase of air voids of asphalt mixtures, the amplitude of the measurement decreased [11]. Therefore, the viscosity of asphalt binders can be distinguished by the value of T2. Aging will cause the change of the viscosity. The aging degree of the asphalt binder can be estimated by LF-NMR according to its change of viscosity.

Blumich et al. [12] demonstrated how to test the heterogeneity of rubber components and pavement nondestructively by a unilateral NMR relaxation method, NMR-MOUSE. Miknis et al. [13] used NMR technology to directly measure and calculate the surface tension of asphalt binders, as well as other interface parameters such as adhesion, diffusion coefficient, and capillary number. Morozov et al. [14] studied the process of asphaltene sediment using flocculant induced by NMR. Helms et al. [15] also applied NMR technology to the replacement of fatty particles and aromatic rings by one other in natural asphalt. In addition, LF-NMR has also made prominent contributions in studying the aging of asphalt binders [16,17,18], rejuvenation [19,20], diffusion of additives [21,22,23], and modification of rubber powders [24,25,26].

Due to the fact that the asphalt binders need to be pre-treated, mainly pre-heated for most tests, the properties of pre-treated samples will differ from their originals. For examples, asphalt samples for AFM tests are a thin film formed onto glass slides from heated drops. By contrast, LF-NMR sample preparation does not require any pre-treatment, which guarantees the microstructure of asphalt to the greatest extent.

The composition and structure of asphalt binders are crucial to their modification. The objective of this study was to investigate the feasibility of using a novel method to identify the aging of modified asphalt binders and its characteristics of modified asphalt binders by NMR. To this end, SBS and CRM with different dosages were used to prepare modified asphalt binder samples. A rolling thin film oven (RTFO) was used for the short-term aging of asphalt samples. The structure and characteristics of all the samples were then examined by LF-NRM and DSR.

## 2. Materials and Methods

### 2.1. Materials

In this study, the particle size of the CRM is 40 meshes, and its properties are shown in Table 1. SBS is YH-791H thermoplastic styrene butadiene rubber, and its properties are shown in Table 2. A virgin asphalt binder from South Korea was used, and its properties are shown in Table 3. Test samples were character numbered; for modified asphalt binders, the dosage of SBS was 2.5%, 3.5%, and 4.5%, respectively, and the dosage of CRM was 6%, 8%, 10%, 12%, and 14%, respectively, as shown in Table 4.

### 2.2. Methods

#### 2.2.1. Modification of Asphalt Binder

When using an SBS modifier, the base asphalt binder was first heated to 165 °C, and then SBS at the amount of the weight of the asphalt, as listed in the Table 3, was added to the asphalt, before stirring for 5~10 min to ensure the SBS was distributed evenly in the base asphalt binder. The asphalt was then sheared with a BME100LT high-shear mixer for 45 min. Finally, the asphalt was stored in an oven at 175 °C for 2 h for further development. When a CRM modifier was used, a JJ-1 precision booster electric low shear mixer was used for mixing for 30 min. The rest of the operations were the same as for SBS modification.

#### 2.2.2. LF-NMR

In this study, an NMR cross-linked density imaging analyzer (VTNR20-010V-I) was used. The magnetic field intensity was 0.3 ± 0.05 t (Tesla). Probe coil diameter was 11 mm. A Carr–Purcell–Meiboom–Gill (CPMG) pulse train was used. The echo time was 0.05 ms, and the number of echoes was 5000. The main frequency of the instrument was 22 MHz, and the receiving bandwidth was 200 KHz. The waiting time was 1000 ms, and the radio frequency delay was 0.08 ms. In LF-NMR, relaxation time, T2, is extremely dependent on test temperature. T2 increases as the temperature rises. However, excessive temperature will lead to the aging of asphalt samples, so it is necessary to choose an appropriate test temperature. Studies have shown that the visibility of the aging of the asphalt binder is more obvious at medium and high temperatures (40~60 °C) than at room temperature [27]. In order to better observe the signal of asphalt binders, the temperature in the hole was set at 60 °C. The samples were tested after 10 min insulation in the hole. Sample preparation steps:

(1) For unaged asphalt binders, at first, heat an unaged asphalt binder of 100 g in a glass bottle to 135 °C. Second, pour the asphalt binder heated into a weighed empty chromatographic bottle with 1.5 mL to 1.0 mL grade. Then, weigh the fully filled chromatographic bottle again. Lastly, calculate the weight of the asphalt sample.
(2) For aged asphalt binders, first, age the asphalt binders By RTFO and PAV, then follow the steps used for (1) for the rest of the steps.

#### 2.2.3. Dynamic Shear Rheometer

The rheological properties of asphalt samples, such as complex shear modulus, G*, phase angle, δ, and anti-rutting factor, G*/sin (δ), were tested at different temperatures (46 °C, 52 °C, 58 °C, 64 °C, 70 °C, 76 °C, and 82 °C) by a MALVERN CVO100 dynamic shear rheometer (DSR). A parallel plate with a diameter and spacing of 25 mm and 1 mm was used, and the loading frequency was 10 HZ.

#### 2.2.4. Aging of Asphalt Binders

According to the Standard Test Methods of Bitumen and Bituminous Mixtures for Highway Engineering (JTP E20-2011), RTFOT (short-term aging of asphalt binders) and PAV (long-term aging of asphalt binders) tests are based on T 0610-2011 and T 0630-2011, respectively. The test temperature of RTFOT was 163 ± 0.5 °C, the test time was 85 min, and the hot air flow rate was 4000 ± 200 mL/min. After RTFOT short-term aging, the original asphalt binders were subjected to PAV long-term aging; the temperature of the PAV test was 100 °C ± 0.5 °C, the pressure was 2.1 ± 0.1 mpa, and the test time was 20 h.

## 3. Results and Discussions

In a given collection time, the shorter the echo time is, the more data points are collected, and the more accurate the fitting of attenuation is [6]. The distribution and signal amplitude of T2 can be determined after data inversion.

### 3.1. T2 of SBS Asphalt Binders Unaged

**Figure 1:**
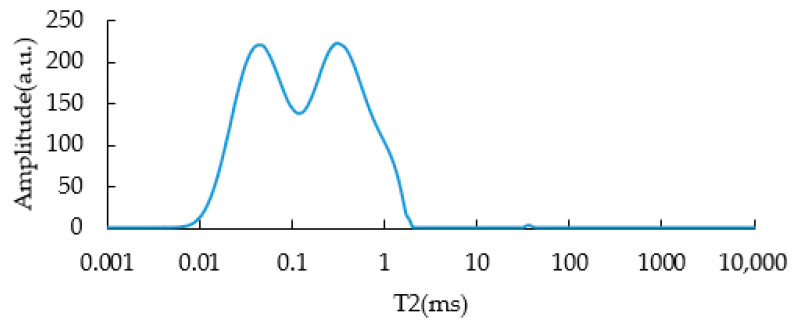
T2 of base asphalt binder unaged.

In order to study the transverse relaxation time, T2, of SBS-modified asphalt binders, the T2 of the virgin asphalt binder before modification must be clearly known (as shown in Figure 1). It can be seen from the figure that the T2 spectrum of the virgin asphalt binder at 60 °C was mainly composed of two wide and fast relaxation peaks (named left-peak and right-peak, respectively), forming an “M” shape relaxation peak with a relaxation time of less than 2.2 ms. On the logarithmic scale of the X axis, the peak times were 0.045 ms and 0.314 ms, respectively.

Traditional NMR theory defines the linear relationship between volume relaxation rate and fluid viscosity. However, for high-viscosity objects such as asphalt binders, the relationship between volume relaxation rate and viscosity is no longer linear because, at high viscosity, T2 decays very fast; this means that the greater the viscosity of the asphalt, the shorter the relaxation time of T2 [28].

In addition, studies have shown that the number of hydrogen atoms per unit mass decreases as the asphalt binder aging increases; therefore, the relative hydrogen index (RHI) is a parameter related to the viscosity and chemistry of asphalt binders. Meanwhile, the differences in the cumulative amplitude among binders with different aging states mainly depend on the hydrogen nucleus to weight ratio. This means that RHI is proportional to the amplitude index of the T2 spectrum. The amplitude index is amplitude/mass, and could be used as an indicator of the oxidation of an asphalt binder [8,29]. Therefore, the relaxation peak in the T2 spectrum can represent the signal strength of the hydrogen nucleus. After being normalized, the stronger the signal, the larger the peak area. Therefore, the number of hydrogen atoms per unit mass (or RHI) is proportional to the total normalized peak area (NPA) of the relaxation peak.

The formula of NPA is shown in Equation (1):

(1) $$\mathrm{NPA}=\frac{A}{m}$$

where A represents the area of peak and m represents mass.

**Figure 2:**
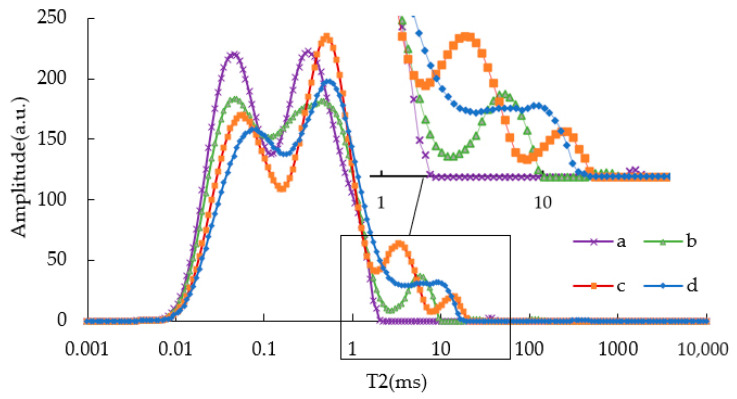
T2 of SBS asphalt binders unaged.

Figure 2 showed the T2 spectra of modified asphalt binders with different SBS content. Firstly, when T2 was within 2 ms, the “M” relaxation peaks appeared in all asphalt samples. With the change of SBS modifier content, the time and area of peaks also changed, accordingly. Through inversion and normalization calculation, it is indicated that when the “M” relaxation peak was formed, the starting and ending time of b-asphalt (2.5%SBS) was 0.002~0.119 ms for the left peak and 0.129~2.801 ms for the right peak, respectively. As the content of the SBS modifier increased up to 4.5%, the starting and ending time was 0.002~0.164 ms for the left peak and 0.178~3.872 ms for the right peak, respectively. The results show that the T2 starting and ending time of modified asphalt binders shifted slightly to the right as SBS content increased, indicating that an increase in the content of SBS prolonged the T2 time of the asphalt binders.

Theoretically, the increase of SBS content will improve the viscosity of asphalt binders, and an increase in the viscosity of an asphalt binder will result in a shorter T2 time. However, the results showed the opposite trend, although the increase in T2 relaxation time is not significant. This may be related to the composite system of SBS and asphalt binders. Menapace et al. [11] studied asphalt binders of asphalt mixtures with different aging degree and air void, and they found that different asphalt binders had different amplitude and relaxation times under the same viscosity. They claimed that the result was caused by the water in the aggregate. For this study, SBS dispersed and expanded in asphalt after high-speed shear to form a composite system. However, the signals of SBS and the base asphalt are different, resulting in different relaxation times of T2 under different SBS dosages, which also leads to a new relaxation peak. For example, in the range of T2 from 2 to 22 ms, b-asphalt had a peak with an ending time of 10.234 ms, c-asphalt had two peaks, with a final ending time being 21.215 ms, and d-asphalt also had two peaks, with the final ending time being 19.564 ms.

**Figure 3:**
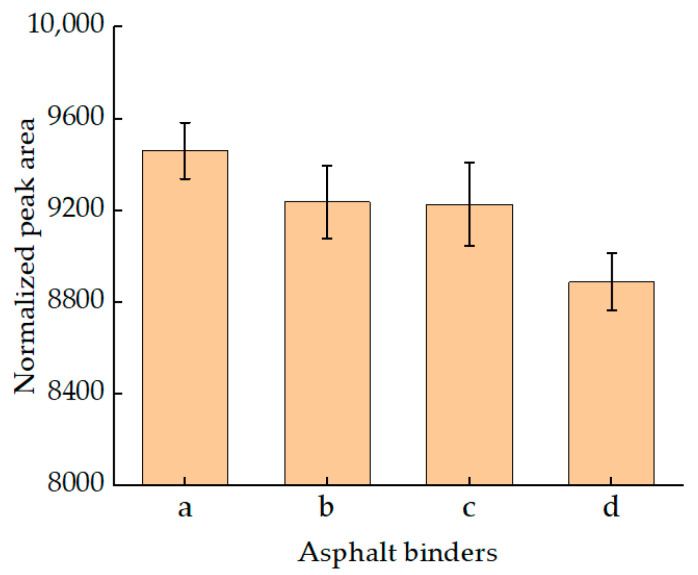
The NPA of unaged SBS asphalt binders.

In the T2 spectrum, the total peak area of an “M” shape was quantitatively analyzed because the trend of the two relaxation peaks was not very uniform. Figure 3 shows the NPA of SBS asphalt binders after inversion calculation. The figure shows that the total relaxation peak area of SBS asphalt binders was smaller than that of virgin asphalt binders. As the content of an SBS modifier increased, the total area of relaxation peak of SBS asphalt binders decreased. That is to say, the viscosity of the SBS asphalt binder was higher than that of the virgin asphalt binder, and the more SBS was added in this study, the higher the viscosity. Strictly speaking, the peak area of the SBS asphalt binder included a peak area after T2 greater than 2 ms, so the peak area of the “M” shape of SBS asphalt binders should be smaller.

### 3.2. T2 of SBS Aged Asphalt Binders

**Figure 4:**
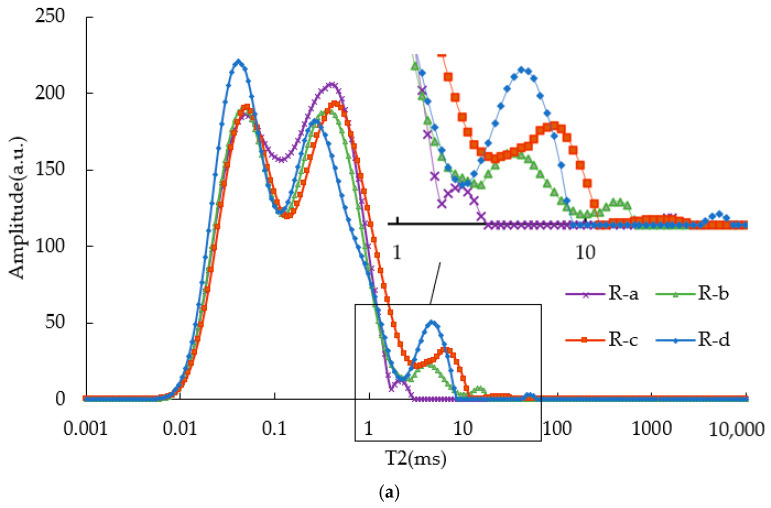

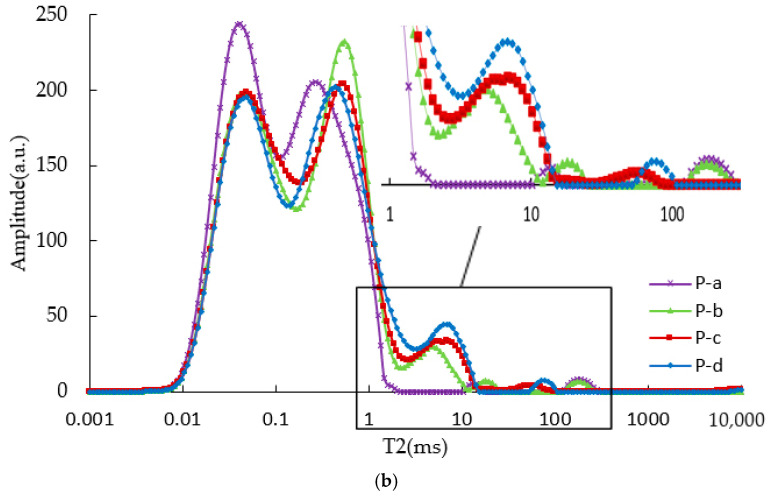
T2 of aged SBS asphalt binders. (**a**) T2 of RTFOT SBS asphalt binders; (**b**) T2 of PAV SBS asphalt binders.

Figure 4 shows the values of T2 of the RTFOT and PAV residuals of SBS-modified asphalt binders with different contents.

It can be seen that the T2 diagrams of these residuals kept the basic “M” shape. However, the left and right peaks of these residuals have irregular changes compared with those of unaged asphalt binders. In addition, the peaks of SBS asphalt binders greater than 2 ms were remarkably weakened, or they even disappeared. This may indicate that aging made some components in the SBS polymer volatilized. As for what is volatilized, further research is needed. It can be seen from Figure 4b that when T2 was greater than 10 ms, PAV residuals had several peaks more than the RTFOT residuals. In particular, when T2 was greater than 100 ms, peaks still appeared in the PAV residuals, but not in the RTFOT residuals. The difference in peak vertex of each asphalt binder was small, and there was no obvious change trend. For R-a, R-b, R-c, R-d, P-a, P-b, P-c, and P-d asphalt binders, the vertexes of the “Left-M” peak are: 0.042 ms, 0.042 ms, 0.057 ms, 0.049 ms, 0.042 ms, 0.049 ms, 0.049 ms, and 0.049 ms, respectively. The vertexes of the “Right-M” peak are: 0.314 ms, 0.435 ms, 0.471 ms, 0.314 ms, 0.267 ms,0.554 ms, 0.511 ms, and 0.435 ms, respectively.

**Figure 5:**
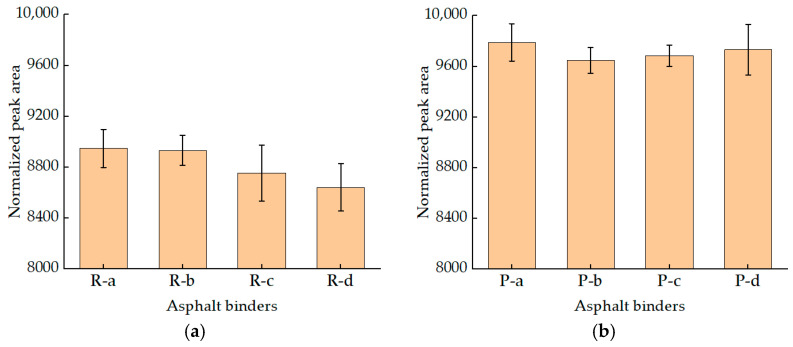
The NPA of aged SBS asphalt binders. (**a**) The NPA of RTFOT SBS asphalt binders; (**b**) The NPA of PAV SBS asphalt binders.

Normalized quantitative analysis was also conducted on the NPA of these residuals. The results are presented using histograms, as shown in Figure 5. As can be seen from Figure 5a, RTFOT aging did not change the overall influence trend of SBS content on NPA. The trend is that, as the content of an SBS modifier increased, the total NPA of SBS asphalt binders decreased. In addition, compared with the unaged asphalt binders, the NPA of RTFOT residuals decreased greatly, which indicated that the viscosity of RTFOT residuals increased; this results was as expected [30].

According to Figure 5b, after PAV testing, the NPA of each asphalt binder was increased, compared with RTFOT residuals and unaged asphalt binders; this result was unexpected. In general, the viscosity of asphalt binders after PAV testing will be further improved. This result can be explained with two possible hypotheses. (1) SBS-modified asphalt binders will have obvious mass loss after PAV testing and will cause the change of the percentage of modified modifier and binder, thus leading to the change of NPA. (2) NPA is not only related to the viscosity of the sample, but also to RHI. Ran studied SBS-modified asphalt after long-term aging and found that, after the aging of SBS-modified asphalt, the proton Har on the aromatic ring decreased, but the content of methyl proton Hα on the saturated carbon chain increased, and the ring structure of Hγ+Hβ increased. The C=C bond of the olefin functional group was broken or recombined [31]. This may lead to the increase of the signal of H and the increase of the area of NPA.

### 3.3. T2 of CRM Asphalt Binders Unaged

**Figure 6:**
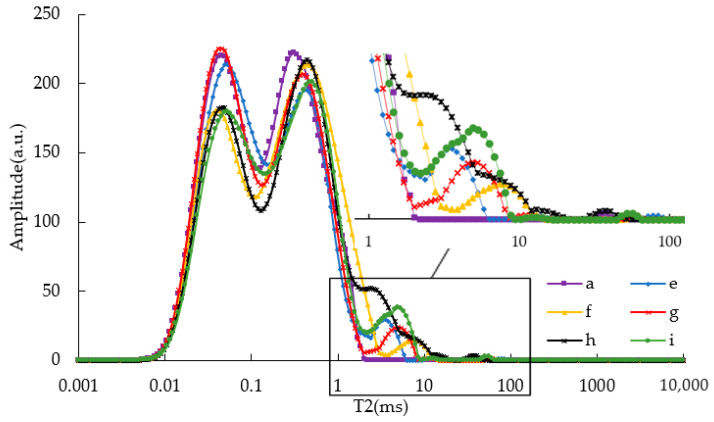
T2 of CRM asphalt binders unaged.

Figure 6 shows the T2 spectrum of CRM asphalt binders, which is also divided into two parts for analysis. When T2 was less than 2 ms, each asphalt binder showed the main peak “M” relaxation peak, and the starting time of each left peak was 0.02 ms. Similar to SBS-modified asphalt binders, the addition of CRM increased the relaxation time. When T2 was greater than 2 ms, multiple relaxation peaks also appeared. Compared with SBS asphalt binders, this part of the peak length of CRM asphalt binders was longer. When T2 was between 2 and 100 ms, three relaxation peaks appeared in each asphalt binder. Their final ending times were as follows: e-asphalt (6%CRM) was 116.232 ms, f-asphalt (8%CRM) was 77.526 ms, g-asphalt (10%CRM) was 71.494 ms, h-asphalt (12%CRM) was 56.072 ms, and i-asphalt (14%CRM) was 65.932 ms. It can be seen that, in general, these relaxation peaks come from CRM modifiers and are shifted to the left as CRM content increased.

**Figure 7:**
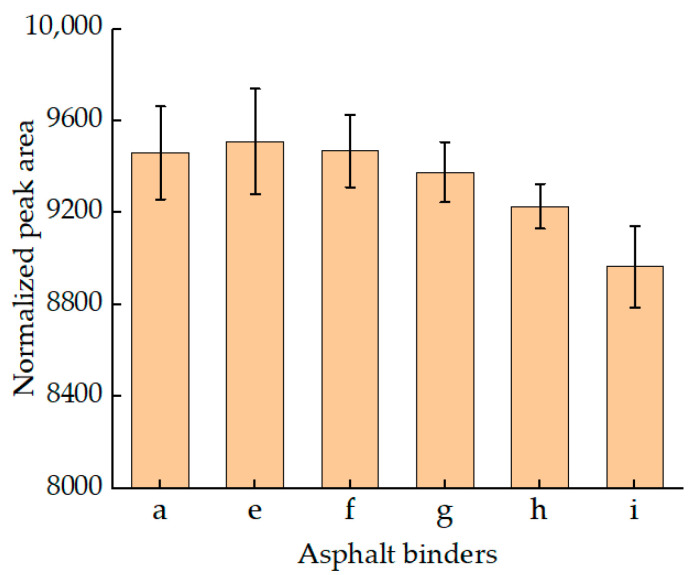
The NPA of CRM asphalt binders unaged.

Normalized inversion calculation was conducted for the relaxation peak in Figure 6 to obtain its total normalized peak area, as shown in Figure 7. For modified asphalts e~i, the more CRM modifiers there were, the smaller the NPA and the higher the viscosity of the modified asphalt binders; this showed the same trend as SBS-modified asphalt binders. Affected by the CRM relaxation peak after 2 ms, the NPA of e~i asphalts will be larger, which may explain why the NPA of e~i asphalt was larger than that of a-asphalt.

### 3.4. T2 of Aged CRM Asphalt Binders

**Figure 8:**
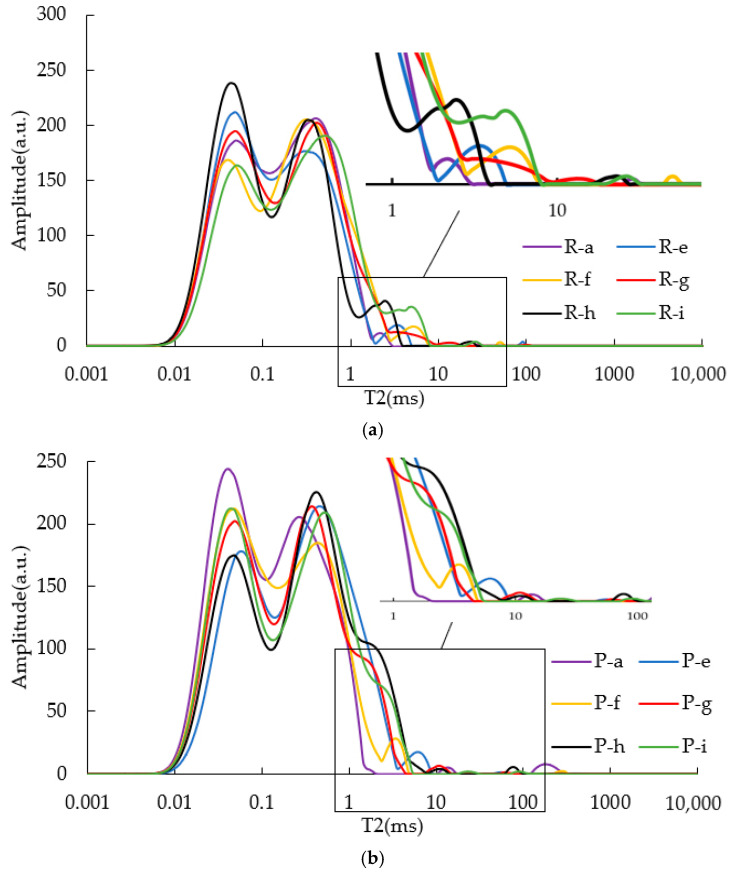
T2 of aged CRM asphalt binders. (**a**) T2 of RTFOT CRM asphalt binders; (**b**) T2 of PAV CRM asphalt binders.

Figure 8 shows the values of T2 of RTFOT and PAV residuals of CRM-modified asphalt binders with different contents. On the whole, the main peak of residuals remained basically unchanged, which was still the “M” shape peak within 2 ms. Similar results were found with SBS asphalt binders: relaxation peaks beyond 2 ms were weakened or even disappeared after being aged. This indicated that some components of the CRM modifier were volatilized by aging.

**Figure 9:**
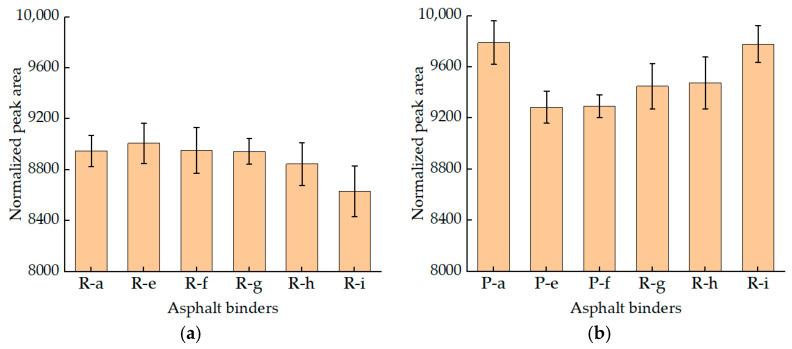
The NPA of aged CRM asphalt binders. (**a**) The NPA of RTFOT CRM asphalt binder; (**b**) The NPA of PAV CRM asphalt binders.

The relaxation peak areas of CRM asphalt binders after aging were normalized (Figure 9). According to Figure 9a, it was found that the peak area of residuals decreased rapidly, which reflected the significant influence of aging on the viscosity of the asphalt binder. Meanwhile, the peak area of asphalt binders decreased with the increase of CRM content, leading to the increase of asphalt viscosity. However, a similar result appears in Figure 9b and Figure 5b. When the CRM content was increased from 6% to 14%, the NPA of the modified PAV residuals were increased. The reason for this result may be similar to that found in SBS-modified asphalt. After long-term aging of modified asphalt, the proportion of modifier and the signal of H changes, leading to the increase of NPA.

To sum up, in the LF-NMR detection, the modification effects of both SBS and CRM modifiers on asphalt binders are, in general, identical in trend and different in degree. The common effect was, without changing the original appearance of major relaxation peaks of asphalt (“M“), their addition reduced the total area of the normalized peaks of the asphalt binders. The viscosity of the asphalt binders increased, and the T2 of “M” peaks were within 2 ms. In addition, aging may lead to volatilization of some components in SBS and CRM modifiers. The difference was that among the peaks other than 2 ms from SBS and CRM modifiers, the relaxation peak T2 of SBS was shorter and tends to shift to the right with the increase of SBS content. In addition, the relaxation peak T2 of CRM is longer and tends to shift to the right with the increase of CRM content. It is worth mentioning that the results of PAV residuals and RTFOT residuals were different.

### 3.5. G*/sin (δ) of Modified Asphalt Binders

The asphalt binder is a typical viscoelastic material with both viscosity and elasticity. In 1987, the American SHRP program introduced dynamic shear rheometers (DSR) into the viscoelastic study of asphalt binders. The rheological properties of all asphalt binders were measured according to the AASHTO T315-04 standard test method. This study mainly analyzed the anti-rutting factor, G*/sin (δ), a parameter to express the deformation resistance of asphalt binders.

**Figure 10:**
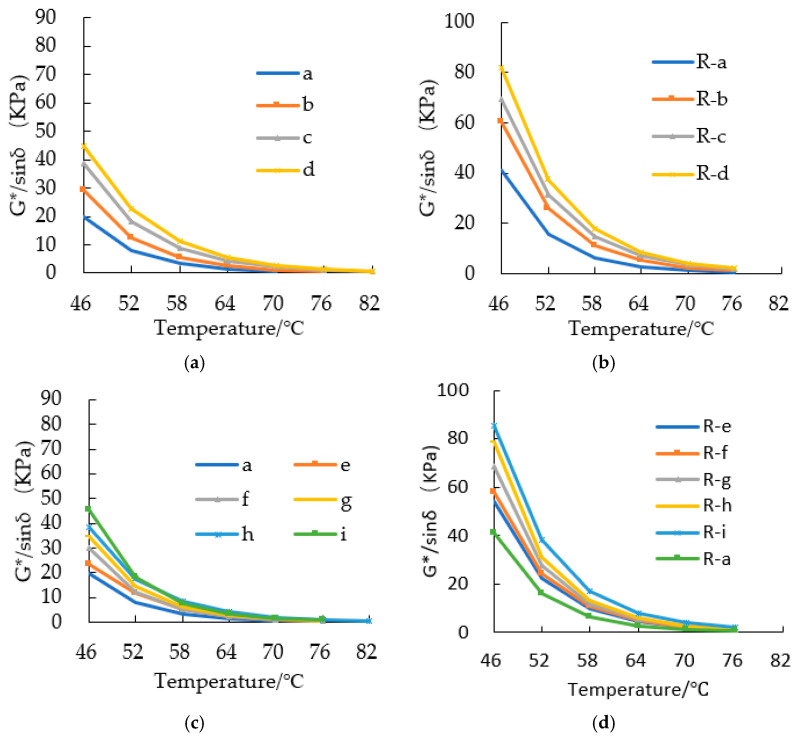
G*/sin (δ) of modified asphalt binders. (**a**) G*/sin (δ) of unaged SBS asphalt binders; (**b**) G*/sin (δ) of aged SBS asphalt binders; (**c**) G*/sin (δ) of unaged CRM asphalt binders; (**d**) G*/sin (δ) of aged CRM asphalt binders.

Figure 10 shows the temperature change trend of asphalt G*/sin (δ) with different amounts of SBS/CRM and different aging degrees. Therefore, it shows that G*/sin (δ) of asphalt binders decreased as the temperature increased. At the same temperature, G*/sin (δ) increased with the increase of SBS/CRM. The G*/sin (δ) of aged asphalt binders were larger than those of un-aged asphalt binders. Before being aged, the G*/sin (δ) of asphalt binders gradually converged when the temperature was higher than 64 °C. After being aged, the coincidence temperature increased to 70 °C.

### 3.6. G*/sin (δ) and NPA of RTFOT Aged Asphalt Binders

**Figure 11:**
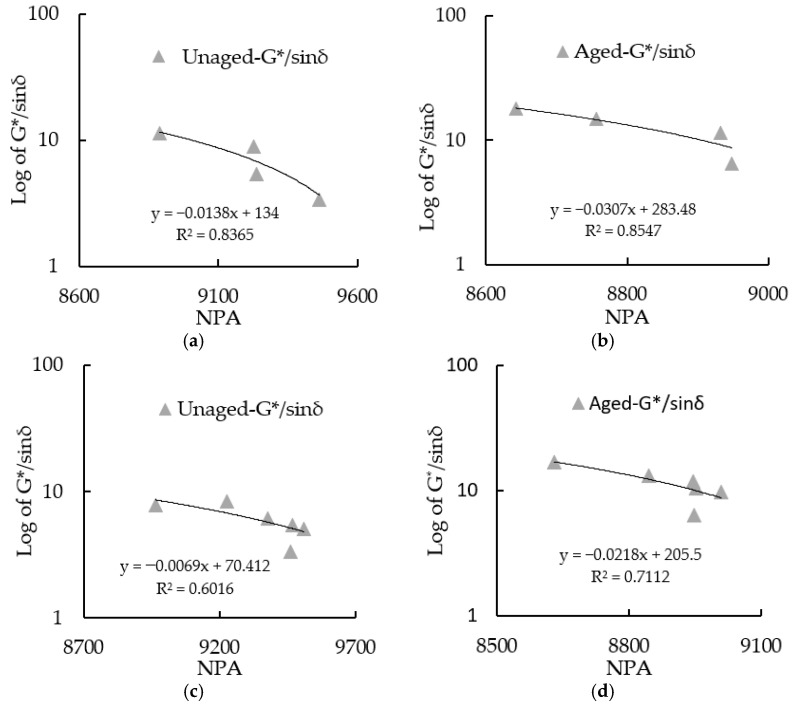
The linear relationship between G*/sin (δ) and NPA. (**a**) G*/sin (δ) and NPA of unaged SBS asphalt binders; (**b**) G*/sin (δ) and NPA of aged SBS asphalt binders; (**c**) G*/sin (δ) and NPA of unaged CRM asphalt binders; (**d**) G*/sin (δ) and NPA of aged CRM asphalt binders.

The G*/sin (δ) of RTFOT residuals at 58 °C is linearly related to NPA. Figure 11a,b shows the SBS asphalt binders before and after being RTFOT aged, respectively, while Figure 11c,d shows the CRM asphalt binders before and after RTFOT was aged, respectively. In all four figures, G*/sin (δ) was inversely proportional to NPA. For SBS asphalt binders, the linear variation extent before RTFOT aging was smaller than RTFOT residuals. However, for CRM asphalt binders, the linear variation extent before RTFOT aging was greater than RTFOT residuals. Therefore, G*/sin (δ) and NPA can be mutually verified. When the NPA was increased, it was seen that G*/sin (δ) decreased, and vice-verse. Therefore, it can be inferred that the rheological change of asphalt binder can be evaluated directly by NPA.

## 4. Conclusions

In this paper, an attempt to characterize the properties of asphalt binders modified by SBS and CRM by LF-NMR was performed. The SBS/CRM asphalt binders before and after being aged were tested by LF-NMR and DSR. The curves between the amplitude and T2 of the measurements were obtained of all the samples modified with different dosages of SBS and CRM. Discussions were presented on the effect of the type of modifiers (i.e., SBS and CRM) and their dosages on the shape and area of those curves. The following conclusions are dr0.awn:

(1) Virgin asphalt binder had two peaks on the curves of amplitude and T2, i.e., “M” shaped, and the T2 time of the “M” shaped peak was within 2.2 ms. Modified asphalt binders also appeared with “M” shaped peaks within 2.2 ms and several peaks beyond 2.2 ms.
(2) When T2 was greater than 2 ms, relaxation peaks appeared in both SBS and CRM asphalt T2 curves, but these peaks did not show up in the virgin asphalt binder. This may be caused by the signal of the modifier itself. Aging of the asphalt binders made peaks weaken and even disappear.
(3) After RTFOT aging, the NPA of modified asphalt binders decreased as their contents of the modifiers increased, and the NPA of modified RTFOT residuals were much smaller than the unaged. RTFOT aging decreased the NPA of asphalt binders, but the results of PAV residuals and RTFOT residuals were different.
(4) G*/sin (δ) increased as aging increased for both modified and unmodified asphalt binders. Moreover, the larger the SBS or CRM content was, the larger the change range was.
(5) G*/sin (δ) and NPA are inversely proportional, inferring that changes in the rheology of asphalt binder can be characterized by directly total normalizing peak area.
(6) All asphalt samples were only studied in the laboratory, but with the popularity of portable NMR devices, it is possible to directly measure the aging of asphalt pavements in situ, so as to achieve the goal of in-situ aging assessment of asphalt pavement over its lifetime.

## Figures and Tables

**Table 1:** Properties of CRM.

Properties	Actual Value	Standard
Moisture/%	0.8	0~2.0
Apparent density/g·cm^−3^	0.36	0.27~0.39
Ash/%	7.4	0~8.5

**Table 2:** Properties of SBS.

Properties	Actual Value
Volatile matter/%	1.0
300% Tensile stress/MPa	2.0
Ash/%	0.2
Elongation at break/%	700
Melt flow rate/g/10 min	0.01–0.50
Hardness	68

**Table 3:** The properties of Shell asphalt binder.

Properties	Actual Value	Standard	Test Method
Penetration (25 °C)/dmm	69	60–80	T0604-2011
Softening point/°C	47.4	46 min	T0606-2011
Ductility (10 °C)/cm	45	20 min	T0605-2011
Ductility (15 °C)/cm	>150	100 min	T0605-2011
Flash point (COC)/°C	>260	260 min	T0611-2011
Solubility in TCE/%m	>99.8	99.5 min	T0607-2011
Wax/%	1.75	2.2 max	T0615-2000
Viscosity (60 °C), Pa·s	215	180 min	T0625-2011
TFOT, 163 °C, 5 h			
PG	PG64-22	PG64-22	AASHTO
Loss by heating/%m	0.01	−0.8~0.8	T0609-2011
Ductility (10 °C)/cm	7	6 min	T0606-2011
Penetration of residue/%m	65	61 min	T0604-2011

**Table 4:** Character number of test samples.

Type of Modifier	-	SBS			CRM				
Dosage of modifier/%	0	2.5	3.5	4.5	6	8	10	12	14
Unaged	a	b	c	d	e	f	g	h	i
RTFOT	R-a	R-b	R-c	R-d	R-e	R-f	R-g	R-h	R-i
PAV	P-a	P-b	P-c	P-d	P-e	P-f	P-g	P-h	P-i
